# The pyroptosis-related gene signature predicts prognosis and reveals characterization of the tumor immune microenvironment in acute myeloid leukemia

**DOI:** 10.3389/fphar.2022.951480

**Published:** 2022-08-10

**Authors:** Tao Zhou, Kai Qian, Yun-Yun Li, Wen-Ke Cai, Sun-Jun Yin, Ping Wang, Gong-Hao He

**Affiliations:** ^1^ Department of Clinical Pharmacy, 920th Hospital of Joint Logistics Support Force of People’s Liberation Army, Kunming, China; ^2^ College of Pharmacy, Dali University, Dali, China; ^3^ Department of Pharmacy, The Second People’s Hospital of Quzhou Zhejiang, Quzhou, China; ^4^ Department of Cardiothoracic Surgery, 920th Hospital of Joint Logistics Support Force of People’s Liberation Army, Kunming, China; ^5^ Research Center of Clinical Pharmacology, Yunnan Provincial Hospital of Traditional Chinese Medicine, Kunming, China

**Keywords:** pyroptosis, acute myeloid leukemia, prognosis, immune microenvironment, TCGA

## Abstract

**Background:** Pyroptosis is a novel inflammatory form of programmed cell death and a prospective target for cancer therapy. Nevertheless, little is known about the association between pyroptosis-related genes (PRGs) and acute myeloid leukemia (AML) prognosis. Herein, we systematically investigated the specific functions and clinical prognostic value of multiple PRGs in AML.

**Methods:** Univariate and LASSO Cox regression analyses based on TCGA and GTEx databases were used to generate the PRG signature, whose predictive efficacy of survival was evaluated using survival analysis, ROC, univariate and multivariate Cox analyses as well as subgroup analysis. The BeatAML cohort was used for data validation. The association between risk score and immune cell infiltration, HLA, immune checkpoints, cancer stem cell (CSC), tumor mutation burden (TMB), and therapeutic drug sensitivity were also analyzed.

**Results:** Six -PRG signatures, namely, *CASP3*, *ELANE*, *GSDMA*, *NOD1*, *PYCARD*, and *VDR* were generated. The high-risk score represented a poorer prognosis and the PRG risk score was also validated as an independent predictor of prognosis. A nomogram including the cytogenetic risk, age, and risk score was constructed for accurate prediction of 1-, 3-, and 5-year survival probabilities. Meanwhile, this risk score was significantly associated with the tumor immune microenvironment (TIME). A high-risk score is characterized by high immune cell infiltration, HLA, and immune checkpoints, as well as low CSC and TMB. In addition, patients with low-risk scores presented significantly lower IC50 values for ATRA, cytarabine, midostaurin, doxorubicin, and etoposide.

**Conclusion:** Our findings might contribute to further understanding of PRGs in the prognosis and development of AML and provide novel and reliable biomarkers for its precise prevention and treatment.

## Introduction

Acute myeloid leukemia (AML) is the second most common type of leukemia diagnosed in adults and accounted for approximately 30% of all adult leukemia cases (Perna et al., 2017), which is primarily characterized by heterogeneity of molecular abnormalities and aberrant accumulation of immature myeloid progenitors in bone marrow and peripheral blood owing to impaired differentiation of hematopoietic progenitors (Sperlazza et al., 2015). The invasive infiltration of AML is mainly represented by a malignant extramedullary infiltration which involves the skin, lymph nodes, liver, spleen, and even central nervous system, and shows poorer prognosis in clinic (Stölzel et al., 2011). In recent years, although the survival and prognosis of AML patients were relatively prolonged with the development and application of molecular targeted therapy and combination therapy in clinical practice, the 5-year survival rate remained at 27% for AML patients over 20 years old and even 69% for patients younger than 20 partially due to the absence of reliable prognostic biomarkers (Ball and Stein, 2019; Schlenk et al., 2019; Zheng et al., 2020). Thus, identifying novel and effective prognostic biomarkers is crucial for improving the AML prognosis and better understanding the pathogenesis of AML.

Recently, much attention was paid to the influences of the tumor microenvironment (TME) on tumorigenesis and development, and alterations in TME components were found at every stage of malignant development in almost all carcinomas (Wu and Dai, 2017; Arneth, 2019). As an important component of TME, the tumor immune microenvironment (TIME) was also found to play prominent roles in tumor cell proliferation, invasion, and metastasis (Fu et al., 2019). Numerous studies showed that TIME was a key determinant of diagnosis and therapeutic response in tumor patients (Xu B. et al., 2021; Yang et al., 2021; Zeng et al., 2021; Wang et al., 2022). However, TIME is complex and variable mainly because of the multiple interaction networks among tumor, immune, stromal, and mesenchymal cells along with various soluble factors and changes in extracellular matrix (ECM) components as well (Wu and Dai, 2017; Xu B. et al., 2021). Therefore, identification of certain potential biomarkers related to TIME would eventually contribute to better understanding of tumor development and further identification of candidate therapeutic targets.

As a highly specific inflammatory programmed cell death, pyroptosis was reported to be significantly cross-correlated with TIME according to previous studies (Orning et al., 2019; Erkes et al., 2020). When persistent inflammation is present, initial activation and assembly of inflammasome started within host cells (Balahura et al., 2020). Subsequently, the caspase was further activated and produced inflammatory cytokines, eventually resulting in pyroptotic cell death (Broz et al., 2010; Moujalled et al., 2021). In recent years, a growing number of studies demonstrated that pyroptosis played crucial roles in pathogenesis and progression of various types of cancers including AML. It was reported that activation of NLR family pyrin domain containing 1 b (NLRP1b) by small molecule inhibitors of serine dipeptidase 8/9 (DPP8/9) induced caspase-1 dependent pyroptosis, which, in turn, suppressed the development of AML (Johnson et al., 2018). Meanwhile, pyridoxine was also found to induce death of primary AML cell in AML patients and prevent disease progression by activating caspase-3/8 and promoting the release of inflammatory factors (Yang et al., 2020). These findings suggested that pyroptosis provided a tumor-suppressive microenvironment and played an immunomodulatory role in AML. However, the prognostic influence of pyroptosis on AML patients was still largely unknown. Moreover, due to technical limitations, most previous studies were limited to a small number of pyroptosis-related genes (PRGs), whereas the involved PRGs might be far more in numbers and their antitumor effects were very likely to interact with each other in a highly coordinated manner. Therefore, a comprehensive analysis regarding the features of TIME cell infiltration mediated by multiple PRGs may provide a relatively whole profile of the function of PRGs and also further insights into the underlying mechanisms of AML occurrence and progression, which, however, has not been investigated so far as we know.

Based on these backgrounds, we, herein, systematically analyzed the differential expression of PRGs and the prognostic value of these genes in clinical practice between AML and normal samples and established an independent prognostic PRG signature. Subsequently, we also explored the relationship between pyroptosis and TIME as well as evaluated the sensitivity of therapeutic drugs for AML patients according to PRG prognostic signature. This study identified reliable prognostic biomarkers for AML patients and provided a novel scientific basis for future immunotherapy in AML.

## Materials and methods

### Acquisition of data

The specific analysis process of the present study is illustrated in Supplementary Figure S1. The RNA sequencing (RNA-seq) data of 151 AML patients’ bone marrow (BM) samples and 755 normal peripheral whole blood samples were acquired from The Cancer Genome Atlas (TCGA) database (https://portal.gdc.cancer.gov/) and the Genotype-Tissue Expression (GTEx) database (https://www.gtexportal.org/). Meanwhile, we obtained the corresponding clinical features of 151 AML patients from TCGA and eventually included 132 samples after excluding 19 samples without survival time. Furthermore, the gene expression profile and relevant clinical characteristic data of 91 AML patients were downloaded from the BeatAML database (Tyner et al., 2018) (http://www.vizome.org/aml/) as a validation cohort. All patients’ clinical features are detailed in Supplementary Table S1. The RNA-seq data of raw count normalized from the aforementioned databases were utilized for differential expression analysis. Then, the count values were converted to transcripts per kilobase million (TPM) values, which were further transformed to log2 (TPM + 1) for subsequent analysis. In addition, the somatic mutation and copy number variation (CNV) data were also retrieved from TCGA database.

### Pyroptosis-related genes

A total of 44 PRGs were retrieved from the GeneCards (https://www.genecards.org/) and previously published literature works (Man and Kanneganti, 2015; Wang and Yin, 2017; Karki and Kanneganti, 2019; Xia et al., 2019), all of which were protein-coding genes. The full details of these genes are listed in Supplementary Table S2. However, as the expression profile of *GSDME* and *PTVK* could not be acquired from the GTEx database, we finally selected 42 PRGs for further analysis. The location of PRGs on the chromosome was plotted by the “RCircos” R package. The “limma” R package was utilized to identify the differentially expressed pyroptosis–related genes (DEPRGs) with a *p* value <0.05 and |log_2_FC| > 0 between tumor and normal tissues. The PRG somatic mutation landscape was presented *via* the “maftools” R packages. The frequencies of genetic amplification and deletion were also summarized. The univariate Cox regression was performed to identify PRGs significantly associated with overall survival (OS) in TCGA cohort. Simultaneously, the Spearman correlation test was applied to evaluate the associations across all PRGs and the comprehensive results were visualized using the “igraph” R package.

### Consensus clustering

Consensus clustering was adopted to identify the distinct pyroptosis-related patterns pertaining to the expression of pyroptosis regulators using k-means algorithms (Hartigan and Wong, 1979). The numbers and stability of clusters were determined by the consensus clustering algorithms of the “ConsensuClusterPlus” R package (Wilkerson and Hayes, 2010). We conducted 1,000 times repetitions to guarantee the classification stability.

### Construction and validation of a prognostic gene signature by prognostic DEPRGs

The significant prognostic-related DEPRGs were presented *via* the “VennDiagram” R package. Subsequently, the least absolute shrinkage and selection operator (LASSO) Cox regression analysis using the “glmnet” R package was applied to screen out the optimal candidate gene combination to construct the prognostic gene signature (Tibshirani, 1997). The optimal value of the penalty parameter *λ* was determined by 10-fold cross-validation based on the minimum criteria. According to the coefficient calculated by LASSO regression and the standardized and normalized TCGA AML expression level, the individual risk score of each AML patient could be calculated using the following formula:

$$Risk\;score=\overset{n}{\underset{i=1}{\sum }}Exp\;i*Coef\;i.$$

Simultaneously, the TCGA AML patients were separated into high- and low-risk categories according to the median risk score. The principal component analysis (PCA) and *t*-distributed stochastic neighbor–embedding (*t*-SNE) analysis were conducted using the “Rtsne” and “ggplot2” R packages to investigate the distribution of various groups in terms of gene expression levels in the constructed model. Thereafter, we performed Kaplan–Meier analysis *via* the “survminer” R package to assess the survival difference between the two categories. The “survival” and “timeROC” R packages were utilized to perform the time-dependent receiver operating characteristic (ROC) curve analysis, which was applied to evaluate the prognostic gene signature’s predictive value. The prognostic significances of gene signature and other clinical characteristics were further investigated using univariate and multivariate Cox regression analyses. Moreover, the same formula and statistical methods were used to further validate the prognostic capacity of the gene signature in the BeatAML cohort.

The chi-squared test was adopted to explore the association of gene signature and clinicopathological characteristics, which was visualized with a heatmap using the “pheatmap” R package. The Wilcoxon signed-rank test and Kruskal–Wallis *H*-test were utilized to compare the risk score among various categories of these clinicopathological characteristics, and the visualization of results was presented *via* the boxplots.

### Establishment of the predictive nomogram

The independent clinical features (cytogenetic risk, age, and risk score) validated using univariate and multivariate Cox regression analyses were enrolled to construct a predictive prognosis nomogram using the “rms” and “survival” R packages. Time-dependent ROC curves for 1, 3, and 5 years were used to assess the performance of the nomogram. The calibration curves for 1-, 3-, and 5-year prediction were utilized to depict the consistency between predicted and actual survival. Furthermore, an alluvial diagram was drawn to show the changes in pyroptosis-related clusters, risk score, age, and cytogenetic risk using the “ggalluvial” R package.

### Functional enrichment analysis of DEGs based on the high- and low-risk groups

AML patients in TCGA cohort were batched into two groups according to the median risk score, respectively. The same approach was also performed in the BeatAML cohort. Afterward, the differentially expressed genes (DEGs) were extracted by utilizing the “limma” R package with the criteria of FDR <0.05 and |log_2_FC| > 1. Gene ontology (GO) and Kyoto Encyclopedia of Genes and Genomes (KEGG) pathway enrichment analyses of the DEGs were performed using the “clusterProfiler” R package (Wu et al., 2021).

### Assessment of the tumor immune environment

We applied the ESTIMATE algorithm to calculate the stromal, immune scores, and ESTIMATE scores of each patient in TCGA and BeatAML cohorts (Yoshihara et al., 2013). Furthermore, considering the significant roles of the immune cell infiltration in the TIME, the infiltrating scores of 22 kinds of human immune cells in each AML sample were computed by the CIBERSORT algorithm with 1,000 permutation. Furthermore, the single-sample gene set enrichment analysis (ssGSEA) algorithm using the “gsva” R package was utilized to calculate infiltration abundance of 29 immune signatures in the AML TIME. Subsequently, we explored the association between the enrichment scores of 22 kinds of immune cells and the risk score or expression levels of the aforementioned identified optimal candidate genes. In addition, the Wilcoxon signed-rank test was applied to estimate the differences in expression of human lymphocyte antigen (HLA) signature and immune checkpoint genes between high- and low-risk groups.

### Analysis of CSC, TMB, and drug susceptibility between high- and low-risk groups

The gene expression–based stemness index was acquired from the previous study (Malta et al., 2018). We explored the correlation between risk score and cancer stemness cell (CSC). Simultaneously, we also computed the tumor mutation burden (TMB) score for each AML sample based on the “maftools” R package and analyzed the differences in the TMB score between high- and low-risk groups, as well as the associations between the TMB score and risk score. To investigate the differences in efficacy of therapeutic drugs in patients between high- and low-risk categories, we calculated the semi-inhibitory concentration (IC50) values of drugs commonly used for the treatment of AML *via* the “pRRophetic” R package.

### Statistical analysis

All statistical analyses were performed by R software (version 4.0.4). Association coefficients were calculated by the Spearman correlation test. Log-rank tests were used for identifying the significance of differences in Kaplan–Meier analysis curves. *p* values of less than 0.05 were considered statistically significant (*p < 0.05) in all analyses.

## Results

### Landscape of expression and genetic alterations of PRGs in AML

**FIGURE 1:**
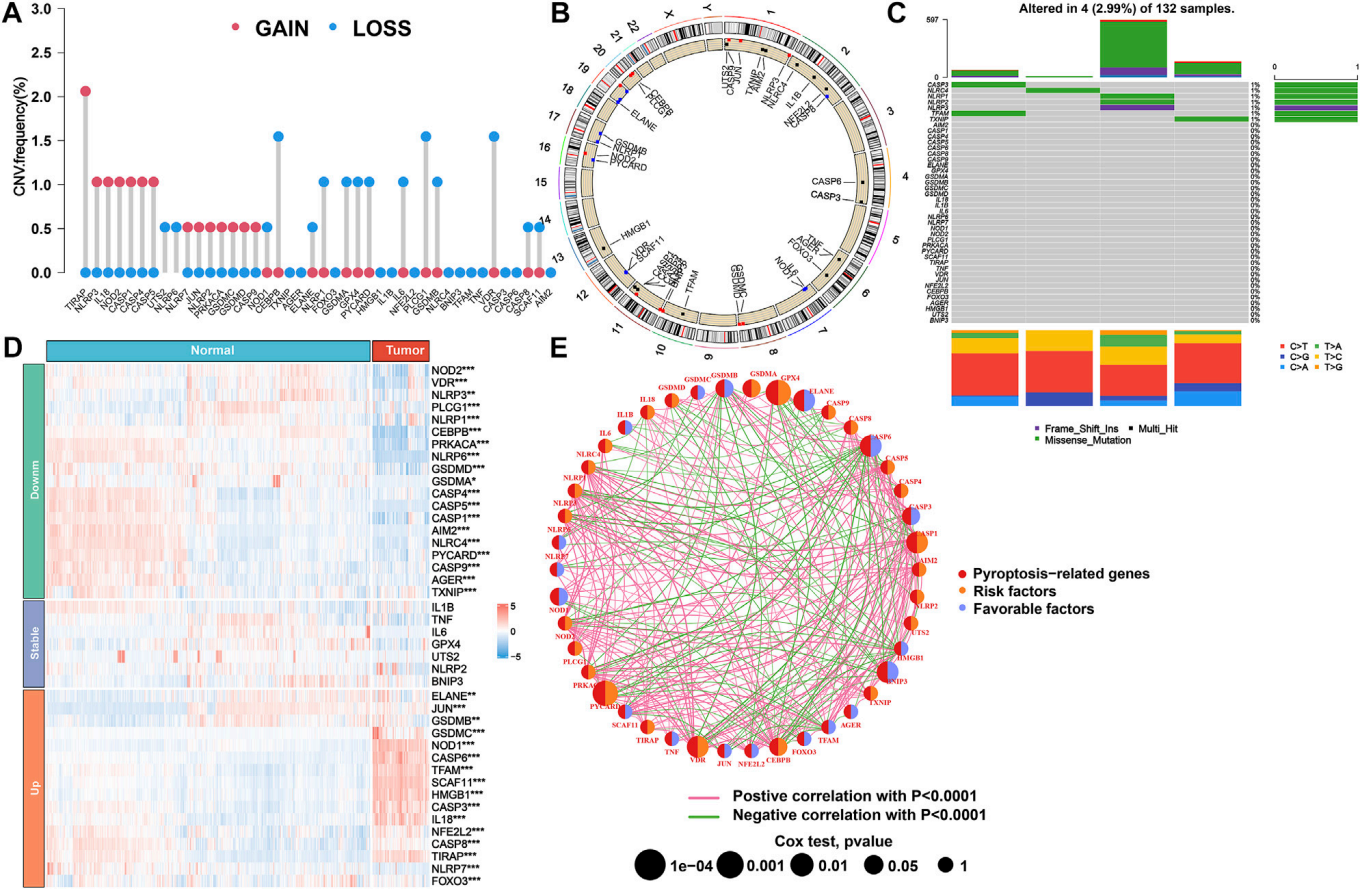
Genetic and transcriptional alteration of PRGs in AML. **(A)** Frequencies of CNV gain, loss, and non-CNV for 42 PRGs in TCGA cohort. **(B)** Locations of CNV alterations in PRGs on 23 chromosomes. **(C)** Mutation frequency of PRGs in TCGA cohort. **(D)** Heatmap of expression patterns of PRGs in normal and tumor samples. **(E)** Network of correlations including PRGs in TCGA cohort. PRGs, pyroptosis-related genes; AML, acute myeloid leukemia; CNV, copy number variant; TCGA, The Cancer Genome Atlas.

This study first summarized the incidence of CNVs and somatic mutations of 42 PRGs in AML. The exploration of CNVs demonstrated prevalent CNV alterations in all 42 PRGs, among which the CNV of *TIRAP* was significantly increased while the CNVs of *CEBPB*, *PLCG1*, and *VDR* were significantly decreased (Figure 1A). Furthermore, the location of CNV variation in the PRGs on their respective chromosomes is displayed in Figure 1B. In the following assessment of the genetic mutation of PRGs in depth, only *CASP3* (1%), *NLRC4* (1%), *NLRP1* (1%), *NLRP2* (1%), *NLRP3* (1%), *TFAM* (1%), and *TXNIP* (1%) showed the genetic mutation in AML patients (Figure 1C). To determine whether these gene variants influence the expression of PRGs in patients with AML, we further calculated the mRNA expression levels of 42 PRG between normal and tumor specimens and then identified the statistically significant DEGs (*p* < 0.05) that correspond to them. As a result, 19 downregulated and 16 upregulated genes were observed in the tumor group (Figure 1D). Subsequently, we discovered that the CNV alteration may be a major factor resulting in perturbations on PRG expression levels. In comparison to normal samples, the expressions of genes with deleted CNVs were found to be significantly decreased in AML samples (such as *CEBPB*, *PLCG1*, and *VDR*), and vice versa (such as *TIRAP*). Based on the aforementioned results, the composite landscape of interaction and correlation of the 42 PRGs and their prognostic value for AML patients was further comprehensively displayed in a pyroptosis network, which showed that 12 pyroptosis genes exhibited a significant prognostic value **(** Figure 1E; Supplementary Tables S3, S4).

### Tumor classification based on the DEPRGs

**FIGURE 2:**
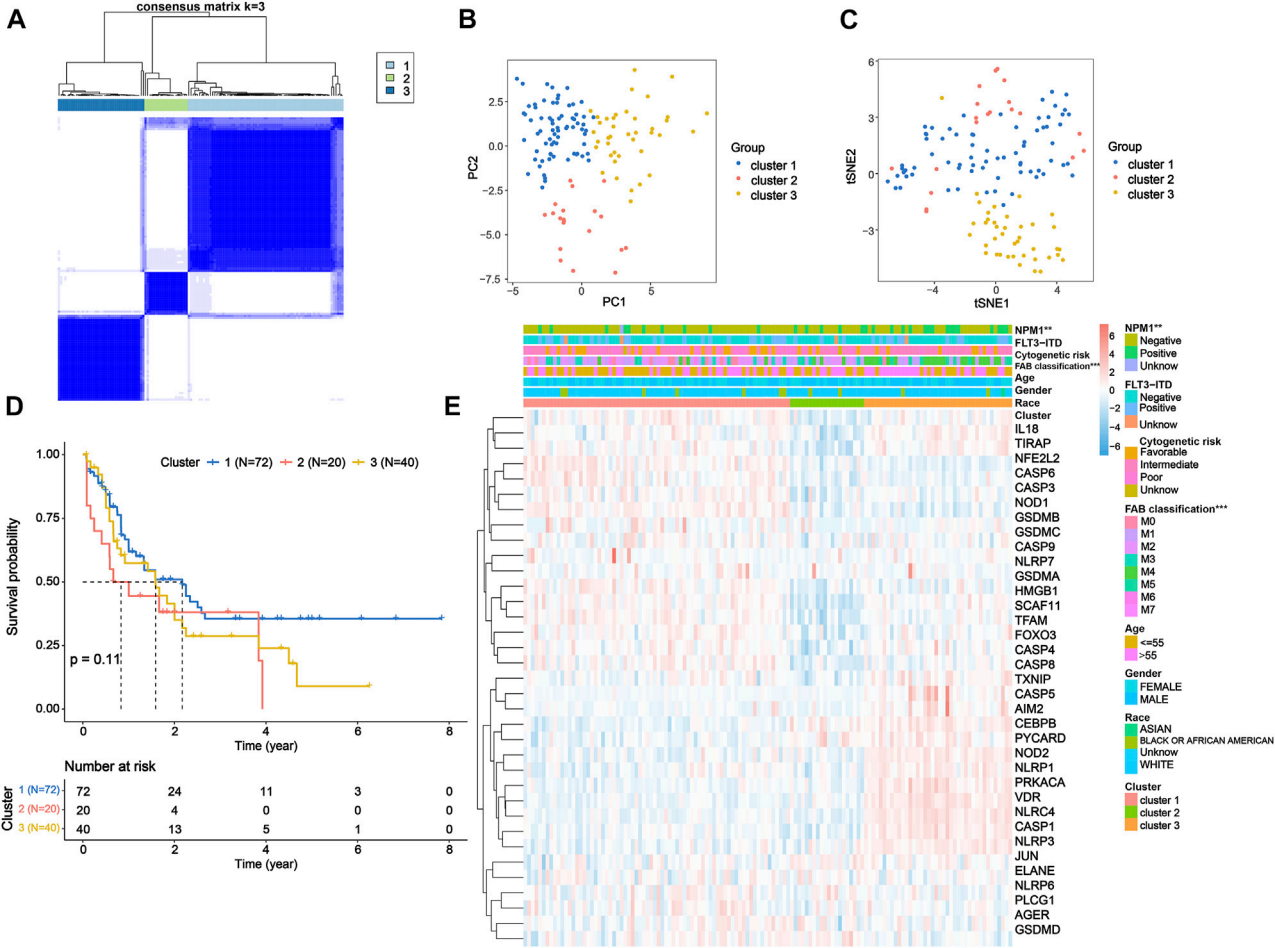
Tumor classification based on the DEPRGs. **(A)** 132 AML patients were classified into three clusters according to the consensus clustering matrix (*k* = 3). **(B)** PCA and **(C)** *t*-SNE analysis exhibiting a remarkable difference in transcriptomes among three clusters. **(D)** Kaplan–Meier OS curves for three clusters. **(E)** Heatmap and clinicopathological features of three clusters. DEPRGs, differentially expressed pyroptosis-related genes; AML, acute myeloid leukemia; PCA, principal component analysis; *t*-SNE, *t*-distributed stochastic neighbor embedding; OS, overall survival.

To further investigate the expression features of the PRGs in AML, we applied the consensus clustering algorithm to classify the patients with AML according to the expression spectrums of 35 DEPRGs (Supplementary Figures S2A–H). We found that *k* = 3 seemed to be the optimum alternative for categorizing the entire cohort into three subtypes (Figure 2A and Supplementary Figures S2I–J), based on which satisfied separation across the three clusters was achieved according to the PCA and *t*-SNE plots (Figures 2B,C). The OS time was also compared between the three groups, but no significant difference was observed (*p* = 0.11, Figure 2D). Furthermore, the heatmap displayed the PRG expression profile and clinicopathological characteristic such as race, gender, age, FAB classification, cytogenetic risk, FLT3-ITD mutation, and NPM1 mutation, with NPM1 mutation (chi-squared test: *p* < 0.01) and FAB classification (*p* < 0.001) demonstrating significant differences among the three groups (Figure 2E).

### Gene signature construction and validation from prognostic DEPRGs

**FIGURE 3:**
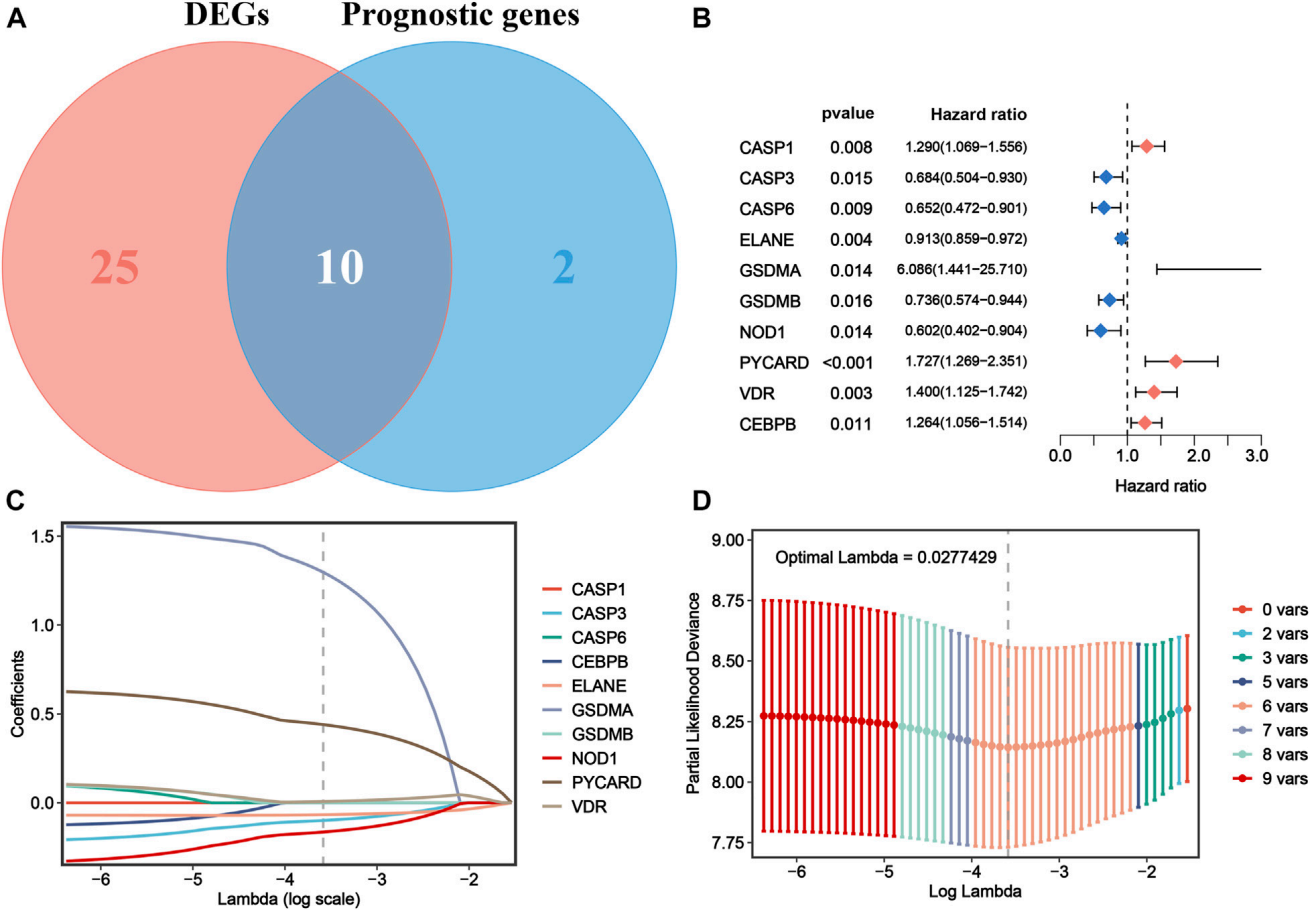
Construction of a PRG signature for AML in TCGA cohort. **(A)** Ten prognostic DEPRGs were identified *via* the Venn diagram. **(B)** Forest plots show the results of univariate Cox analysis of OS for 10 prognostic DEPRGs. **(C)** LASSO regression of the 10 prognostic DEPRGs. **(D)** Cross-validation for turning the parameter selection in the LASSO regression. PRG, pyroptosis-related gene; AML, acute myeloid leukemia; TCGA, The Cancer Genome Atlas; DEPRGs, differentially expressed pyroptosis-related genes; OS, overall survival; LASSO, the least absolute shrinkage and selection operator.

**FIGURE 4:**
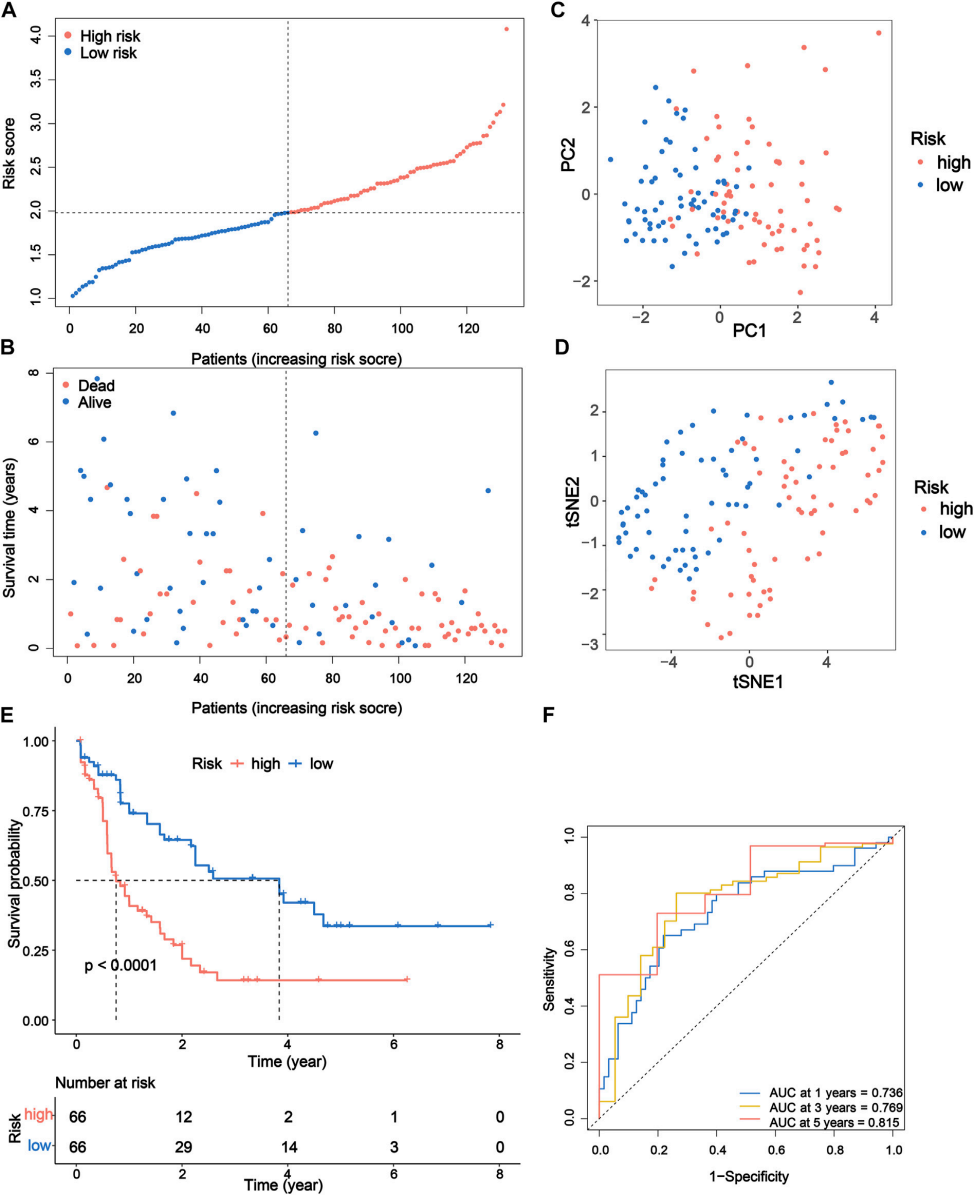
Prognostic study of TCGA cohort *via* the PRG signature model. **(A)** Risk scores and **(B)** survival status of AML patients. **(C)** PCA and **(D)** *t*-SNE analysis showing the different gene expression of samples. **(E)** Kaplan–Meier OS curves for high-risk and low-risk groups. **(F)** The 1-, 3-, and 5-year ROC curve to predict the survival status. TCGA, The Cancer Genome Atlas; PRG, pyroptosis-related gene; PCA, principal component analysis; *t*-SNE, *t*-distributed stochastic neighbor embedding; OS, overall survival; ROC, receiver operating characteristic.

To further explore the prognostic value of the DEPRGs in AML patients, the gene signature was constructed. As shown in Figure 3A, 12 of 42 PRGs showed a significant prognostic value according to univariate Cox regression analysis, among which 10 genes were overlapped between DEPRGs and prognostic genes and were identified as the prognostic DEPRGs. The specific prognostic value of 10 genes is further depicted in Figure 3B and Supplementary Table S5 with five risk and five favorable factors, respectively. Subsequently, based on 1,000 times 10-fold cross-validation in LASSO Cox regression analysis, the minimum of the *λ* value was selected as the optimum *λ* value (0.0278). Then, six of 10 genes with not-zero coefficients were screened to construct the gene signature according to the optimum *λ* **(** Figures 3C,D). The risk score of each patient is computed as follows: risk score = (−0.0993153386641694 × expression of *CASP3*) + (−0.0669072839409446 × expression of *ELANE*) + (1.29694083393916 × expression of *GSDMA*) + (−0.164405100033389 × expression of *NOD1*) + (0.440274519545682 × expression of *PYCARD*) + (0.00707428724075096 × expression of *VDR*). Then, the patients were separated into the high-risk group (*n* = 66) and low-risk (*n* = 66) group according to the median risk score (Figure 4A). The distribution of the risk score indicated that the OS status in the high-risk category was significantly worse than that in the low-risk category (Figure 4B). Further PCA and *t*-SNE analyses showed identifiable dimensions between the high-risk and low-risk categories (Figures 4C,D). Meanwhile, the Kaplan–Meier survival curves demonstrated significantly superior OS in patients with low scores than those with high scores (*p* < 0.0001; Figure 4E). Furthermore, time-dependent ROC analysis also revealed that this gene signature exhibited a favorable prognostic performance with AUCs of 0.736, 0.769, and 0.815 at 1-, 3-, and 5-year, respectively (Figure 4F).

To verify the capability of the 6-gene signature, the BeatAML dataset was downloaded as an external validation cohort. We calculated the risk scores of patients with the same formula and stratified patients into high-risk (*n* = 45) and low-risk (*n* = 46) groups (Supplementary Figure S3A). Similarly, patients in the two groups of the BeatAML cohort were distributed in various directions based on the PCA and *t*-SNE analyses (Supplementary Figures S2C–D). Similar to the results of TCGA cohort, patients with low scores were found to have significantly longer OS and a favorable prognostic value (*p* = 0.0034) in the validation cohort (Supplementary Figures S3B,E). In addition, the results of 1-, 3-, and 5-year ROC curves also possessed relatively higher AUC values (0.654, 0.800, and 0.659), indicating that the signature had excellent predictive capability for survival of AML patients (Supplementary Figure S3F).

### Independent prognostic value of the 6-gene signature and evaluation of clinical characteristics

**FIGURE 5:**
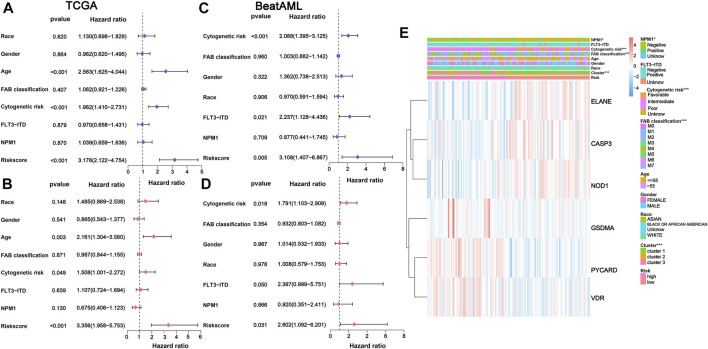
Univariate and multivariate Cox regression analyses for the risk score. **(A)** Univariate and **(B)** multivariate Cox regression of the risk score and other clinical characteristics associated with OS in the TCGA cohort. **(C)** Univariate and **(D)** multivariate Cox regression of the risk score and other clinical characteristics associated with OS in the BeatAML cohort. **(E)** Heatmap for association between clinicopathologic characteristics and risk groups. OS, overall survival.

We performed univariate and multivariate Cox analyses to assess the possibility of the risk score functioning as an independent prognostic factor. The results of univariate Cox regression analysis demonstrated that the risk score was a significant independent predictor of poor survival in both TCGA (*p* < 0.001, HR = 3.176, 95% CI = 2.122–4.754; Figure 5A) and BeatAML cohorts (*p* = 0.005, HR = 3.108, 95% CI = 1.407–6.867; Figure 5C). Multivariate analysis also revealed that the risk score was a critical prognostic factor for AML patients in both cohorts after accounting for other confounders (*p* < 0.001, HR = 3.356, 95% CI = 1.958–5.753 for TCGA cohort and *p* = 0.031, HR = 2.602, 95% CI = 1.092–6.201 for the BeatAML cohort; Figures 5B,D).

The chi-squared test of correlations between the risk subgroups and clinicopathological features indicated that NPM1 mutation (*p* < 0.05), cytogenetic risk (*p* < 0.001), FAB classification (*p* < 0.001), and PRG clusters (*p* < 0.001) were significantly associated with risk subgroups (Figure 5E). Subsequently, the Wilcoxon signed-rank test was applied to compare the differences in risk scores across respective ;groups of the aforementioned clinicopathological characteristics, also demonstrating a remarkable relationshipof these clinicopathological characteristics with risk scores (Supplementary Figures S4A–D).

In addition, we further assessed whether the risk score could still maintain a good prediction of survival across different subgroups. The results showed that low-risk patients also showed a better prognosis than high-risk patients in all subgroups, with statistically significant results in the following subgroups: race (white: *p* < 0.0001), gender (male: *p* = 0.018; female: *p* = 0.0001), age (≤55 years: *p* = 0.024; >55 years: *p* = 0.00045), FAB classification (M0: *p* = 0.017; M2: *p* = 0.011), cytogenetic risk (intermediate: *p* = 0.036), NPM1 mutation (positive: *p* = 0.0051; negative: *p* = 0.0029), and FLT3-ITD mutation (positive: *p* = 0.034; negative: *p* = 0.0019) (Supplementary Figures S5A–R).

### Development of a prognostic nomogram for AML

**FIGURE 6:**
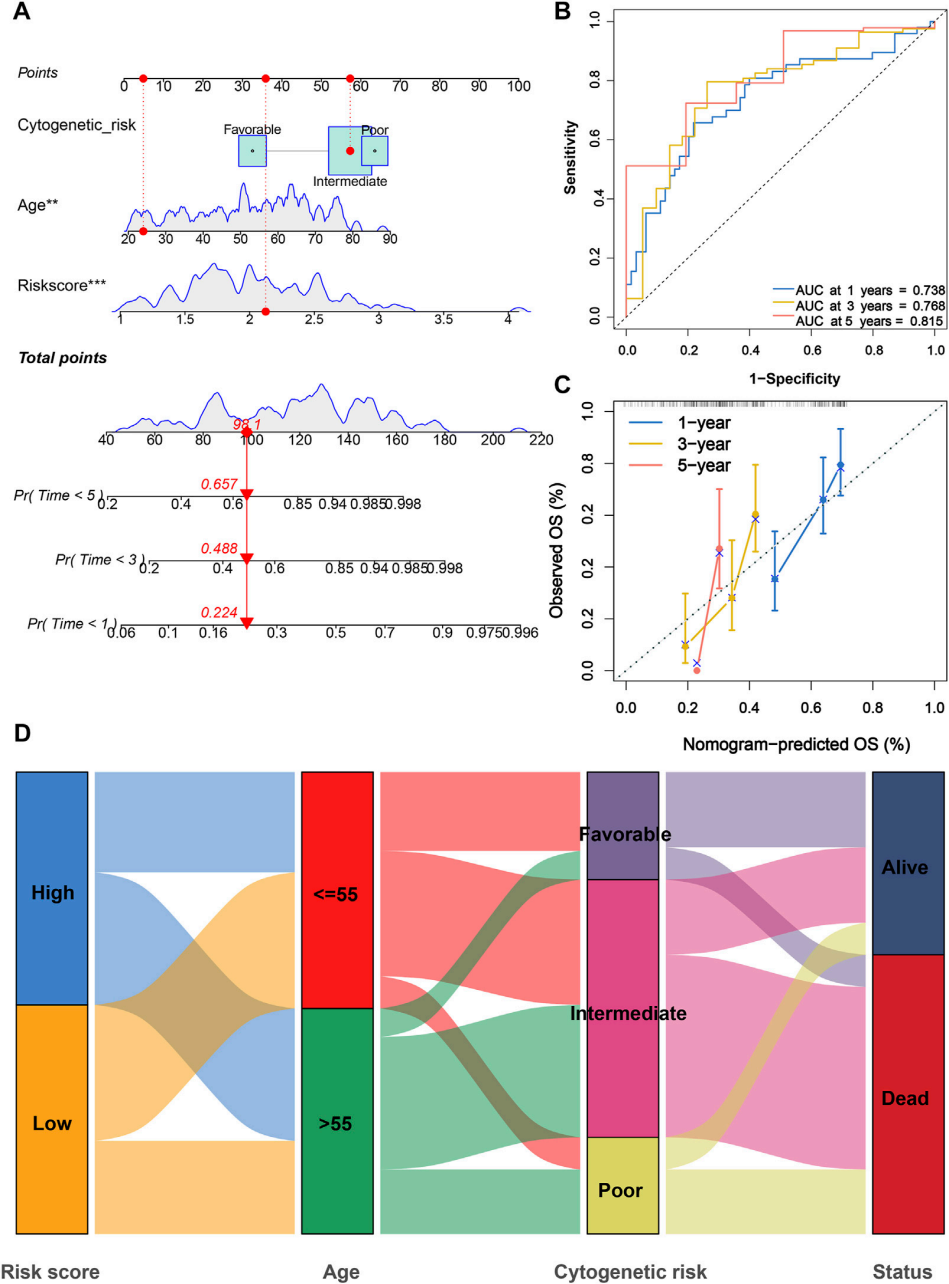
Construction and evaluation of a PRG signature-based nomogram. **(A)** Nomogram incorporating cytogenetic risk, age, and risk score was constructed to predict 1-, 3-, and 5-year survival probabilities. **(B)** ROC curves for 1-, 3-, and 5-year were used to assess the performance of nomogram. **(C)** Calibration curves for 1-, 3-, and 5-year prediction were utilized to depict the consistency between predicted and actual survival. **(D)** Alluvial diagram of subgroup distributions with different risk scores and survival outcomes. PRG, pyroptosis-related gene; ROC, receiver operating characteristic.

In consideration of the fact that the risk score alone was not sufficient to predict OS in AML patients, a nomogram incorporating the risk score and clinicopathological features was constructed to forecast 1-, 3-, and 5-year OS of AML patients according to the significant results of multivariate Cox regression analysis (Figure 6A). Furthermore, ROC curves indicated that this nomogram exhibited a good prognostic performance with AUCs of 0.738, 0.768, and 0.815 at 1-, 3-, and 5-year, respectively (Figure 6B). The subsequent calibration plot showed the proposed nomogram operated in a manner consistent with an ideal model (Figure 6C). Moreover, the alluvial diagram was applied to visualize variations in the aforementioned characteristics of AML patients (Figure 6D).

### Functional annotation of the 6-gene signature

**FIGURE 7:**
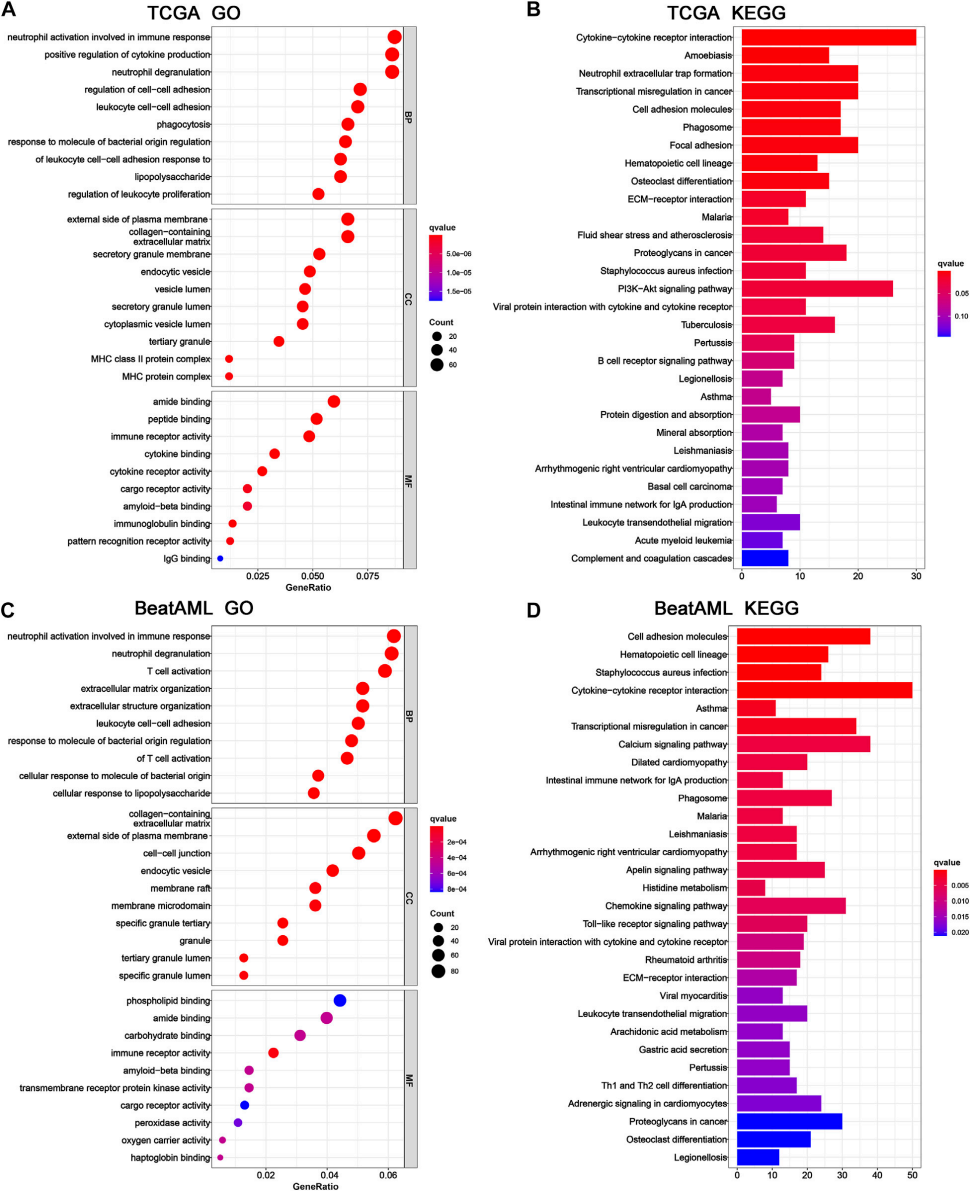
GO and KEGG functional enrichment between high- and low-risk groups. Top 10 results of **(A)** GO and **(B)** KEGG pathway enrichment of DEGs among high- and low-risk groups in TCGA cohort. Top 10 results of **(C)** GO and **(D)** KEGG pathway enrichment of DEGs among high- and low-risk groups in the BeatAML cohort. GO, Gene Ontology; KEGG, Kyoto Encyclopedia of Genes and Genomes; DEGs, differentially expressed genes.

To further investigate the potential biological functions and pathways of the 6-gene signature, the DEGs across the high-risk and low-risk categories were applied to perform GO and KEGG analyses. The specific details of results were presented in Supplementary Tables S6, S7. Furthermore, these DEGs of TCGA cohort were significantly enriched in biological processes and molecular functions with immunity, such as neutrophil activation involved in the immune response, leukocyte cell–cell adhesion, regulation of leukocyte proliferation, immune receptor activity, immunoglobulin binding, and IgG binding (Figure 7A). Several of these biological processes and molecular functions were validated in the BeatAML cohort, including neutrophil activation involved in the immune response, leukocyte cell–cell adhesion, and immune receptor activity **(** Figure 7C). The results of KEGG analysis also showed enrichment of immune-related pathways, which included cytokine–cytokine receptor interaction, phagosome, viral protein interaction with cytokine and cytokine receptor, intestinal immune network for IgA production, and leukocyte transendothelial migration in both TCGA and BeatAML cohorts (Figures 7B,D). Moreover, pathways regarding B-cell receptor signaling and Th1 and Th2 cell differentiation were also found in TCGA (Figure 7B) and BeatAML cohorts (Figure 7D), respectively. In addition, several cancer-related pathways were simultaneously identified in both cohorts, such as transcriptional misregulation in cancer, hematopoietic cell lineage, ECM-receptor interaction, and proteoglycans in cancer. These results revealed that the pyroptosis-related 6-gene signature was significantly associated with cancer progression and particularly possessed an important influence on the immunoregulation of TME.

### Tumor immune microenvironment analysis of 6-gene signature

**FIGURE 8:**
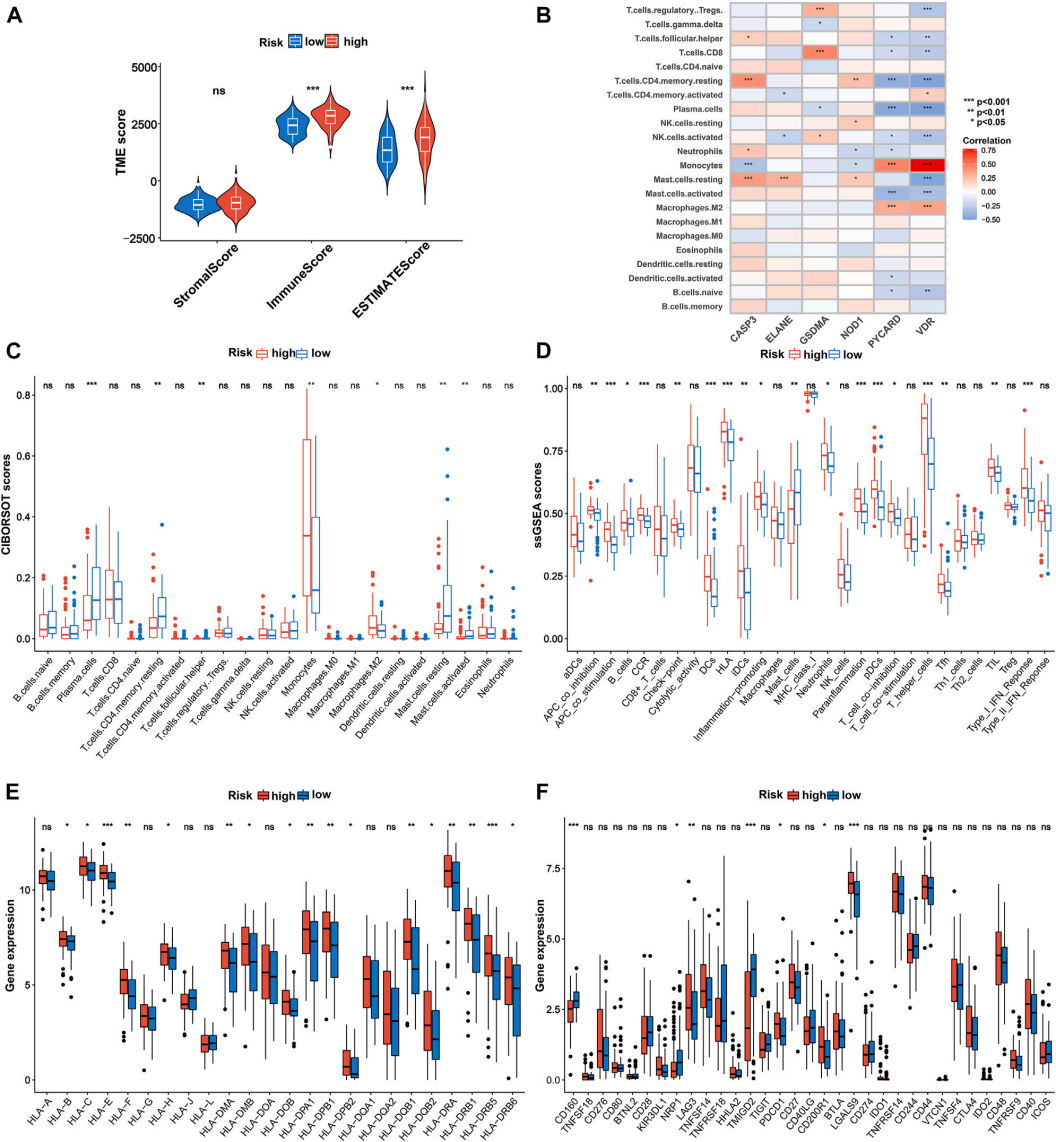
Immune characteristics analysis in TCGA cohort. **(A)** Associations between risk score and both immune and stromal scores. **(B)** Correlations between the abundance of immune cells and six genes in the proposed signature. Comparison of the enrichment scores of **(C)** 22 kinds of immune cells and **(D)** 29 types of immune signatures between high- and low-risk groups. **(E)** Expression of HLA in the high- and low-risk groups. **(F)** Expression of immune checkpoints in the high- and low-risk groups. *p* values are shown as: ns, not significant. *p = < 0.05; **p = < 0.01; ***p = < 0.001. HLA, human lymphocyte antigen.

Next, we further explored the association between the pyroptosis-related gene signature and tumor immune microenvironment and observed that the low-risk patients were significantly correlated with inferior immune and ESTIMATE scores in the TCGA cohort (Figure 8A). Subsequently, based on the CIBERSORT algorithm, we compared the distribution of 22 kinds of immune cells in diverse risk subgroups. A significant difference in the distribution of immune cells was observed in high-risk patients with superior infiltration of monocytes and M2 macrophages, but inferior infiltration of plasma cells, resting memory CD4^+^ T cells, follicular helper T cells, activated mast cells, and resting mast cells (Figure 8C and Supplementary Table S8). Concurrently, we analyzed the connection between the risk score and the infiltration score of immune cells, which further suggested that the risk score was significantly associated with the six kinds of immune cells (Supplementary Figure S6). We also evaluated the relationship between the six pyroptosis-related genes in the proposed signature and abundance of immune cells. The results showed that partial immune cells were significantly associated with the six genes (Figure 8B). Furthermore, comparisons of 29 immune signatures provided by the ssGSEA algorithm revealed that high-risk patients exhibited higher infiltration scores of APC co-inhibition, APC co-stimulation, B cells, CCR, checkpointa, DCs, HLA, iDCs, increased inflammation , neutrophils, parainflammation, pDCs, T-cell co-inhibition, T helper cells, Tfh, TIL, and type I IFN response, whereas lower infiltration scores of mast cells (Figure 8D and Supplementary Table S9) indicated higher immune infiltration among high-risk AML patients.

Considering that HLA-related genes play a critical role in regulating the immune response, we then compared the expression of HLA-related genes between different subgroups and observed that most of the HLA-related genes were upregulated in the high-risk group (Figure 8E). Furthermore, we investigated the correlation between 33 immune checkpoints and the 6-PRG signature. Figure 8F demonstrated that high-risk patients exhibited significantly higher expression of *PDCD1*, *CD200R1*, *CAG3*, and *LGALS9* as well as lower expression of *CD160*, *NRP1*, and *TMIGD2* compared with low-risk patients. In addition, similar results were also observed in the BeatAML cohort (Supplementary Figures S7, S8; Supplementary Tables S10, S11).

### Analysis of CSC index, TMB, and drug susceptibility

**FIGURE 9:**
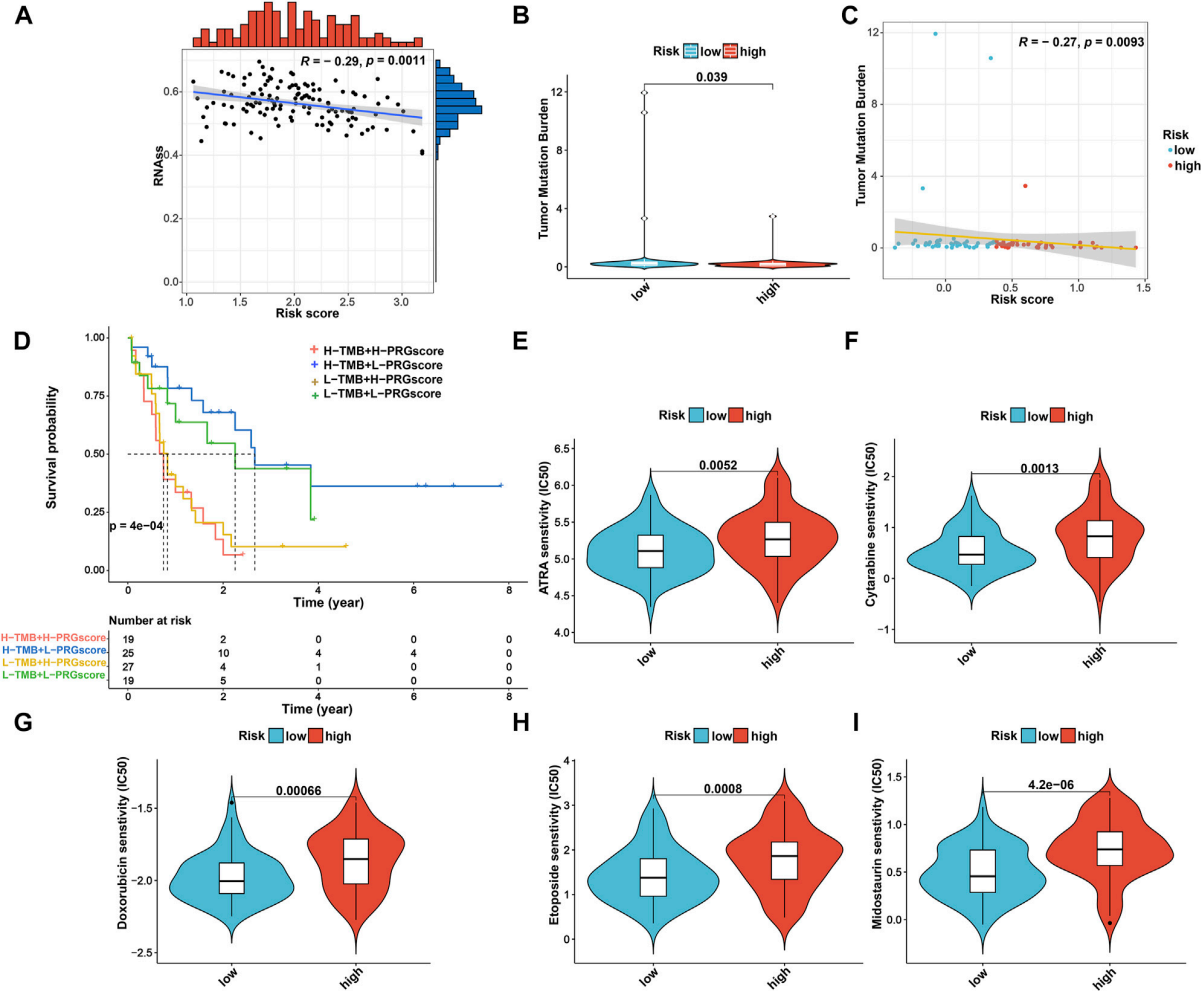
Comprehensive analysis of the risk score in AML. **(A)** Associations between the risk score and CSC index. **(B)** Comparison of TMB between high- and low-risk groups. **(C)** Spearman correlation analysis of risk score and TMB. **(D)** Kaplan–Meier OS curves for different TMB and risk score subgroups. **(E–I)** Relationships between risk score and therapeutic drugs of AML sensitivity. AML, acute myeloid leukemia; CSC, cancer stemness cell; TMB, tumor mutation burden; OS, overall survival.

Considering that the CSC index and TMB play a critical role in the pathogenesis and immunotherapy of AML (Eppert et al., 2011; Snyder et al., 2014), we further explored the potential correlation between them and the PRG signature. As shown in Figure 9A, the risk score was negatively associated with the CSC index (*R* = −0.29, *p* = 0.0011), suggesting that AML cells with a lower risk score exhibited more significant stem cell characteristics and a lower degree of cell differentiation. Meanwhile, we also found that TMB of the low-risk group was significantly higher than that of the high-risk group (*p* = 0.039; Figure 9B) and was negatively associated with the PRG risk score (*R* = −0.25, *p* = 0.019; Figure 9C). Furthermore, the Kaplan–Meier survival curve for combining the PRG risk score and TMB revealed significant differences in survival outcomes and patients with high TMB and low PRG risk scores exhibited a more pronounced survival advantage (Figure 9D).

Moreover, given the impact of drug susceptibility on patients with AML, we further selected the drugs currently used in the treatment of AML to assess the sensitivity of these drugs to patients in both high-risk and low-risk subgroups. Interestingly, we observed that patients in the low-risk group presented significantly lower IC50 values for ATRA, cytarabine, midostaurin, doxorubicin, and etoposide than those in the high-risk group (Figures 9E–I). Therefore, patients with a low PRG risk score might exhibit better treatment benefits when administrating these drugs. Nevertheless, the effect of these drugs in the treatment of AML patients remains to be further proven in future clinical studies.

## Discussion

AML is a rapidly progressive hematologic tumor with poor prognosis, and its development can be influenced by multiple factors (Coombs et al., 2016), among which genes associated with programmed cell death (PCD), such as autophagy-related genes and ferroptosis-related genes, were demonstrated to serve as reliable prognostic biomarkers for AML (Fu et al., 2021; Shao et al., 2021). However, studies regarding the role of PRGs in AML are restricted to individual PRG and have never been investigated systematically and comprehensively. In this study, we detected the global alterations in PRGs at transcriptional and genetic levels in AML and identified a total of 42 currently available PRGs, the majority of which were differentially expressed between AML and normal samples and were associated with prognostic of this disease. Subsequently, we first constructed a reliable and valid PRG signature for AML based on six PRGs. In both training and validation cohorts, the PRG signature exhibited robust capabilities in predicting survival outcomes in AML patients. Furthermore, patients with low and high PRG scores showed significantly different clinicopathological features. In addition, univariate and multivariate Cox regression analyses also indicated that the PRG signature was an independent prognostic factor. These findings confirm that the present PRG signature can be used as a potentially reliable prognostic biomarker in AML patients with various clinicopathological features.

Pyroptosis, a novel form of PCD, is activated *via* the classical caspase-1 inflammasome or non-classical caspase-4, caspase-5, and caspase-11–mediated pathways (Tang et al., 2020). Recently, the dual roles of pyroptosis in tumor progression gained substantial attention. On one hand, it was reported that inflammatory molecules released from cancer cells underwent pyroptosis and gradually converted the surrounding normal cells into cancer cells by altering the microenvironment, which in turn promoted tumor development. On the other hand, induction of tumor cell pyroptosis was also found to inhibit tumor progression and was demonstrated to be a potential therapeutic target for drug development (Tang et al., 2020). This situation raised great uncertainties regarding the exact functions of pyroptosis in different kinds of tumors. Furthermore, as a matter of fact, previous studies paid much more attention to the roles of PRGs in solid carcinomas [e.g., hepatocellular carcinoma (Deng et al., 2022), breast cancer (Xu D. et al., 2021), lung adenocarcinoma (Lin W. et al., 2021), and colon adenocarcinoma (Luo et al., 2021)] with few studies, however, focusing on non-solid tumor such as AML. Therefore, the present study provided a novel signature featuring six PRGs (*CASP3*, *ELANE*, *GSDMA*, *PYCARD*, *VDR*, and *NOD1*) that exactly and independently predict OS in AML patients, which will further contribute to driving the progress of individualized prevention and treatment of AML.

Among these PRGs, caspase-3 (*CASP3*) is an important member of the caspase family, whose activation degrades structural and functional proteins within cells, thereby inducing cell death (Yuan et al., 1993; Jiang et al., 2020). In the present study, we further revealed that this gene was a favorable predictor of survival outcome and was associated with increased sensitivity to chemotherapeutic drug–induced pyroptosis in AML, which was in accordance with the previous study indicating that *CASP3* activated by chemotherapeutic drugs initiated pyroptosis (Wang et al., 2017). Moreover, as one of the primary serine proteases secreted by neutrophils, *ELANE* is another known promoter of pyroptosis, which activates inflammatory factors such as *TNF-α*, *IL-1β*, and *IL-18*, and induces neutrophils to develop pyroptosis (Kambara et al., 2018; Mirea et al., 2020). Consistently, our study demonstrated that the expression of *ELANE* was significantly higher while the neutrophil infiltration score was remarkably lower in the low-risk group than that in the high-risk group, which was very likely to be ascribed to its pyroptosis activating effect in neutrophils. In addition, the following three genes (*GSMDA*, *PYCARD*, and *VDR*) that were previously identified as possible executors of pyroptosis and usually exhibited tumor-suppressive effects (Ding et al., 2016; Šutić et al., 2019; Ling et al., 2022) were also identified and included in the present PRG signature, further confirming its reliability.

Interestingly, as a cytoplasmic pattern recognition receptor, *NOD1* was initially identified as a cancer-promoting factor and might cause tumor recurrence and metastasis, resulting in a poorer prognosis through pyroptosis (Fernández-García et al., 2022; Nomoto et al., 2022). However, in our research, *NOD1* showed a significant cancer suppressive effect and acted as a protective factor against AML. This discrepancy may be attributed to the specialized tumor microenvironment in non-solid tumors and also the antitumor immune activity generated by the combined action of multiple PRGs although the exact mechanism still needs to be further explored.

Previous studies showed that the pro-inflammatory effects of pyroptosis are strongly associated with the regulation of the TIME (Tsuchiya, 2021). This study hence further evaluated the association between the risk score of PRG signature and TIME and found that patients in the high-risk group showed significantly higher immune scores than those in the low-risk group. The abundance of infiltration of tumor immune cells also differed between the high- and low-risk groups. Compared to the high-risk group, resting CD4^+^ memory T cells and follicular helper T cells, both of which were well acknowledged to exert an important antitumor immune response (Watanabe, 2021), infiltrated at higher levels in the low-risk group; whereas tumorigenesis-, angiogenesis- and immune suppressing-related cell types, such as M2 macrophages, monocytes, antigen-presenting cells, and dendritic cells (Nahas et al., 2019; Fu and Song, 2021), showed higher infiltration levels in the high-risk group. Furthermore, this study also found that patients with high-risk scores showed a worse prognosis than those with low-risk scores. These findings indicated that the immunosuppressive microenvironment played important roles during the genesis and development of AML. In fact, it was reported that the formation of an immunosuppressive microenvironment usually prevented the clearance of tumor cells by tumor killer cells, resulting in an increased risk of malignant progression and death (Fridman et al., 2022). Therefore, treatment targeting the immunosuppressive microenvironment may be a more effective and feasible strategy for patients with a poor prognosis of AML.

In addition, we found that most HLA-related genes and *PDCD1* expressed at higher levels in the high-risk group, which was in accordance with the current increasing evidence regarding solid tumors suggesting that more HLA presentation increased the recognition of tumor-associated antigens in HLA and in turn increased the success of immune checkpoint inhibitor therapy (Rizvi et al., 2015; Lin W. Y. et al., 2021). Therefore, patients with high-risk scores for AML might benefit more from immunotherapy, especially with immune checkpoint inhibitors PD-1.

Although we analyzed the effects of pyroptosis on the prognosis of AML and the immune microenvironment as comprehensively as possible, the following points remained inadequate. First, all analyses in this study were performed based on retrospective data from public databases, and large prospective studies and additional *in vivo* and *in vitro* experimental studies are still needed to confirm our findings. Second, the proposed gene signature model was validated only through public databases. Therefore, further clinical trials are still necessary to confirm its clinical utility. In addition, the potential mechanisms of the present six key genes used for model construction need to be further explored in order to better understand the tumorigenesis and development of AML. Finally, subgroup analyses for ethnicity were not performed in our study mainly due to the limited availability of current original data, which should also be validated in future studies based on additional risk models.

In summary, we comprehensively analyzed the expression and genetic changes of PGRs in AML, their prognostic value in the clinic, their important role in TIME, and constructed a signature consisting of six PRGs, which was confirmed to be an independent predictor for OS in AML patients. The results of this study will contribute to further understanding of the important role of pyroptosis in the prognosis and development of AML and provide novel and reliable biomarkers for its precise prevention and treatment.

## Data Availability

The original contributions presented in the study are included in the article/Supplementary Material; further inquiries can be directed to the corresponding author.
