# Supplementary material for: The pyroptosis-related gene signature predicts prognosis and reveals characterization of the tumor immune microenvironment in acute myeloid leukemia
Source: Front Pharmacol. 2022 Aug 10;13:951480. doi: 10.3389/fphar.2022.951480 (PMC9399441; doi:10.3389/fphar.2022.951480)
Supplement: Supplementary file 1 [file DataSheet1.ZIP › Supplementary Table S1.docx]

Table S1 The clinical characteristics of TCGA and BeatAML

|  | **TCGA (Training cohort)** | **BeatAML (Validation cohort)** | ***P*** |
| --- | --- | --- | --- |
|  | ***N=132*** | ***N=91*** |  |
| Gender: |  |  | 0.859 |
| Female | 61 (46.2%) | 44 (48.4%) |  |
| Male | 71 (53.8%) | 47 (51.6%) |  |
| Age: |  |  | NA |
| <=55 | 67 (50.8%) | NA |  |
| >55 | 65 (49.2%) | NA |  |
| Race: |  |  | <0.001 |
| Asian | 1 (0.76%) | 3 (3.30%) |  |
| Black | 0 (0.00%) | 1 (1.10%) |  |
| Black or African American | 11 (8.33%) | 0 (0.00%) |  |
| HispNative | 0 (0.00%) | 8 (8.79%) |  |
| Unknow | 2 (1.52%) | 0 (0.00%) |  |
| White | 118 (89.4%) | 79 (86.8%) |  |
| FAB classification: |  |  | <0.001 |
| M0 | 12 (9.09%) | 6 (6.59%) |  |
| M1 | 32 (24.2%) | 7 (7.69%) |  |
| M2 | 32 (24.2%) | 7 (7.69%) |  |
| M3 | 14 (10.6%) | 9 (9.89%) |  |
| M4 | 27 (20.5%) | 26 (28.6%) |  |
| M5 | 12 (9.09%) | 32 (35.2%) |  |
| M6 | 2 (1.52%) | 0 (0.00%) |  |
| M7 | 1 (0.76%) | 2 (2.20%) |  |
| NOS | 0 (0.00%) | 2 (2.20%) |  |
| Cytogenetic risk: |  |  | <0.001 |
| Favorable | 30 (22.7%) | 39 (42.9%) |  |
| Intermediate | 73 (55.3%) | 27 (29.7%) |  |
| Poor | 27 (20.5%) | 25 (27.5%) |  |
| Unknow | 2 (1.52%) | 0 (0.00%) |  |
| FLT3-ITD: |  |  | 0.069 |
| Negative | 90 (68.2%) | 73 (80.2%) |  |
| Positive | 38 (28.8%) | 18 (19.8%) |  |
| Unknow | 4 (3.03%) | 0 (0.00%) |  |
| NPM1: |  |  | 0.925 |
| Negative | 100 (75.8%) | 68 (74.7%) |  |
| Positive | 31 (23.5%) | 23 (25.3%) |  |
| Unknow | 1 (0.76%) | 0 (0.00%) |  |
| Status: |  |  | 0.094 |
| Alive | 52 (39.4%) | 47 (51.6%) |  |
| Dead | 80 (60.6%) | 44 (48.4%) |  |

Abbreviation: FAB: French-American-British, FLT3-ITD: FMS-like tyrosine kinase 3-internal tandem duplication, NPM1: Nucleophosmin 1
