# Supplementary figures and images for: The pyroptosis-related gene signature predicts prognosis and reveals characterization of the tumor immune microenvironment in acute myeloid leukemia
Source: Front Pharmacol. 2022 Aug 10;13:951480. doi: 10.3389/fphar.2022.951480 (PMC9399441; doi:10.3389/fphar.2022.951480)

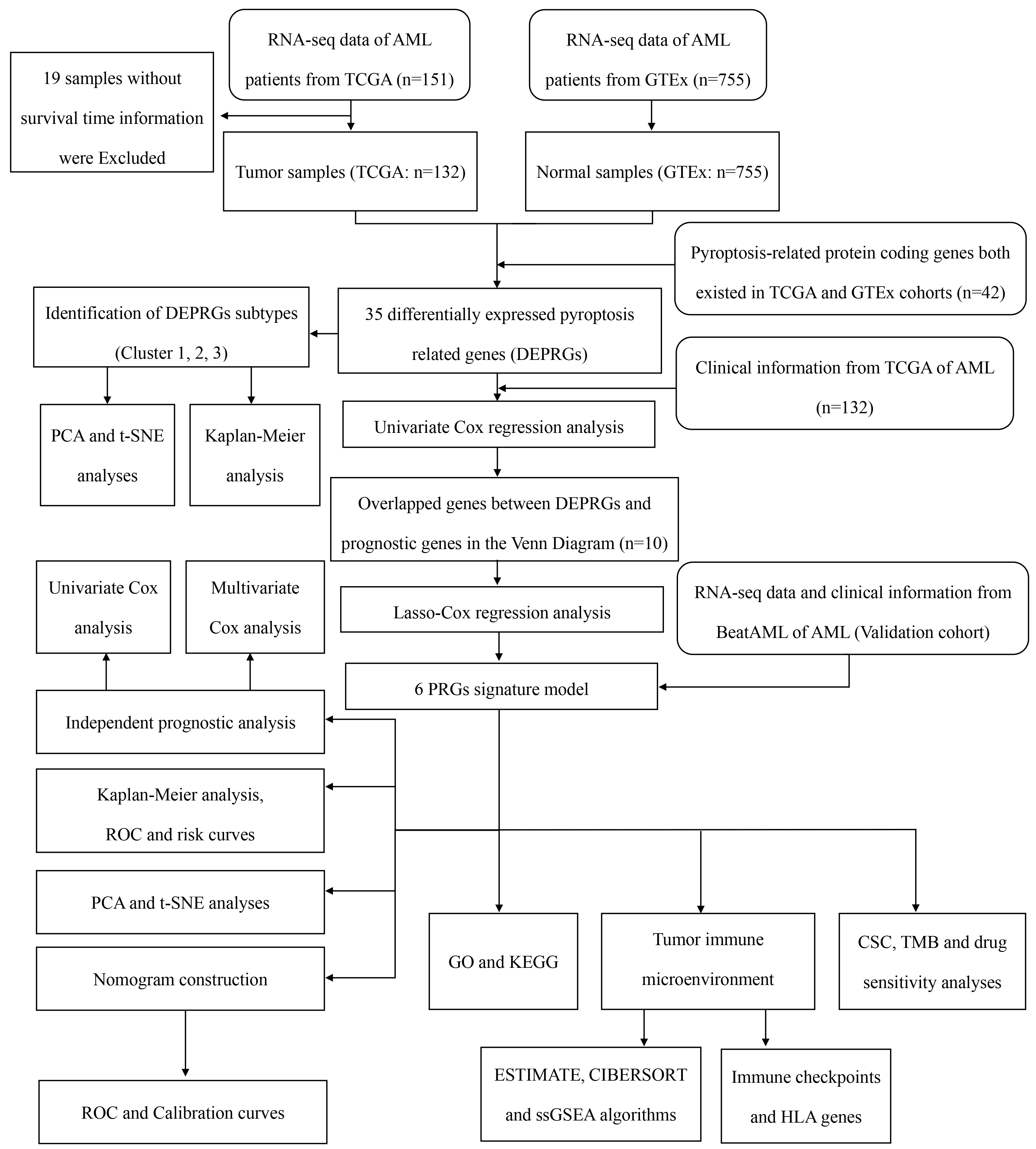

Supplement: Supplementary file 1 [file DataSheet1.ZIP › Supplementary Figure S1.tif]

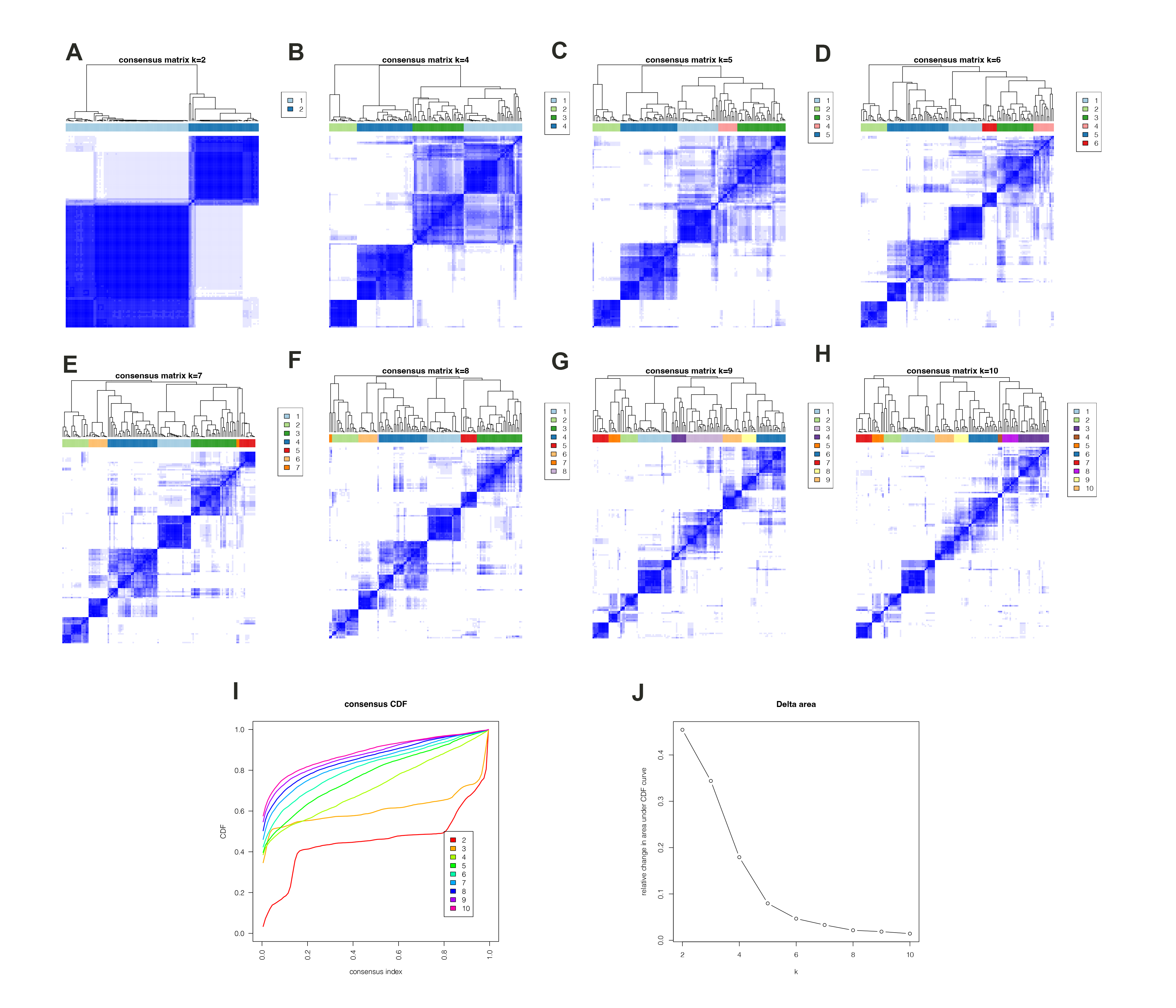

Supplement: Supplementary file 1 [file DataSheet1.ZIP › Supplementary Figure S2 .tif]

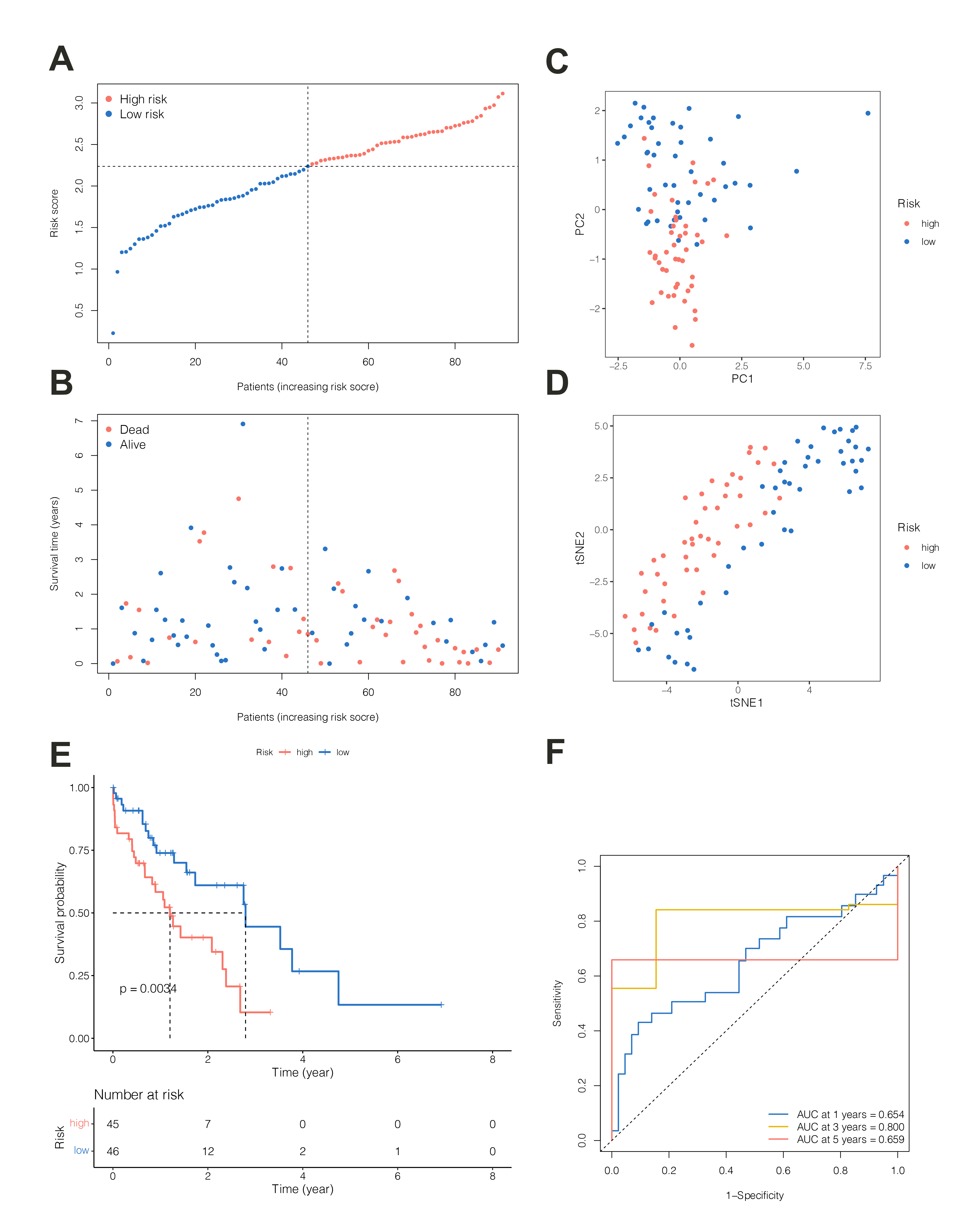

Supplement: Supplementary file 1 [file DataSheet1.ZIP › Supplementary Figure S3 .tif]

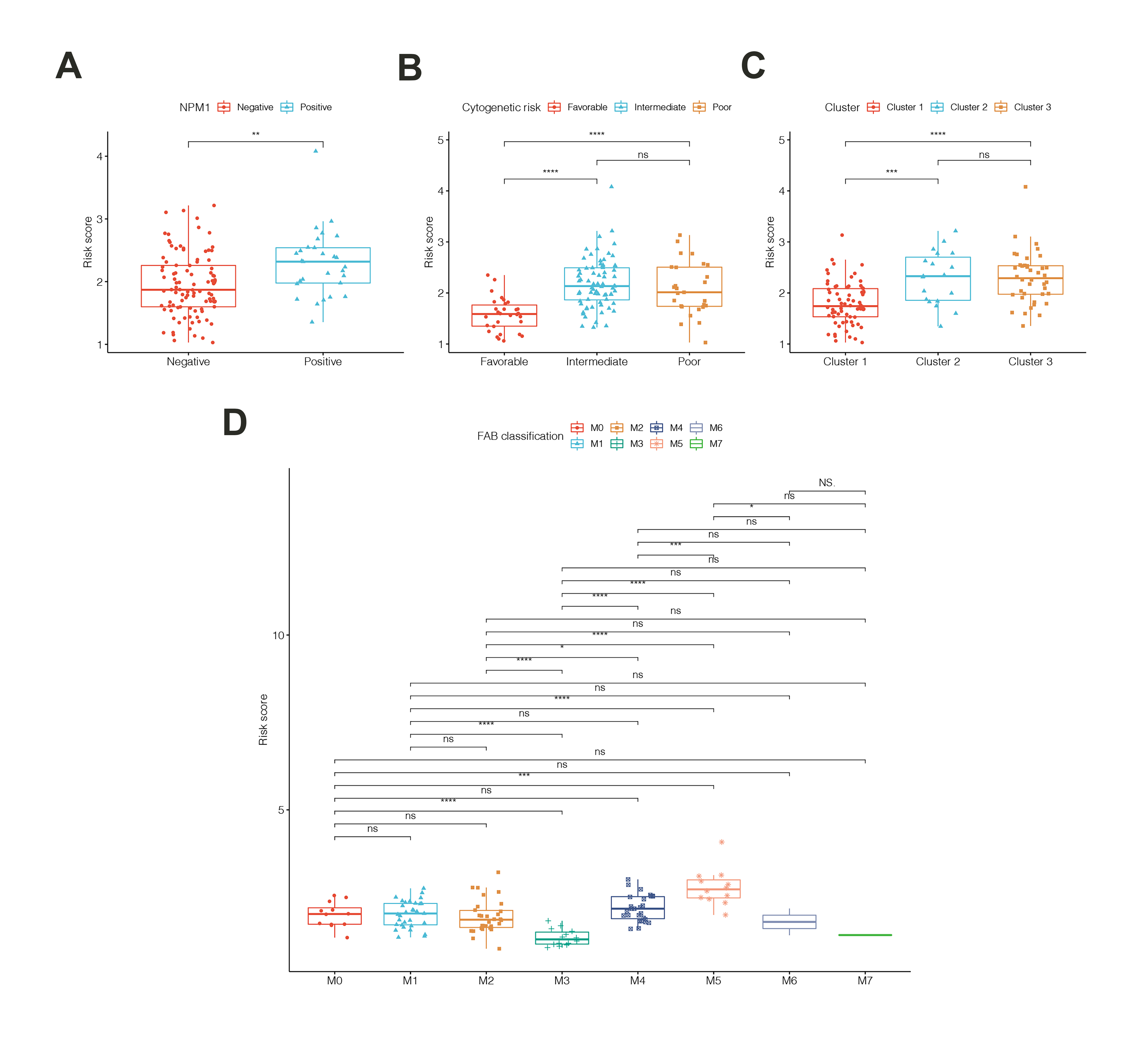

Supplement: Supplementary file 1 [file DataSheet1.ZIP › Supplementary Figure S4 .tif]

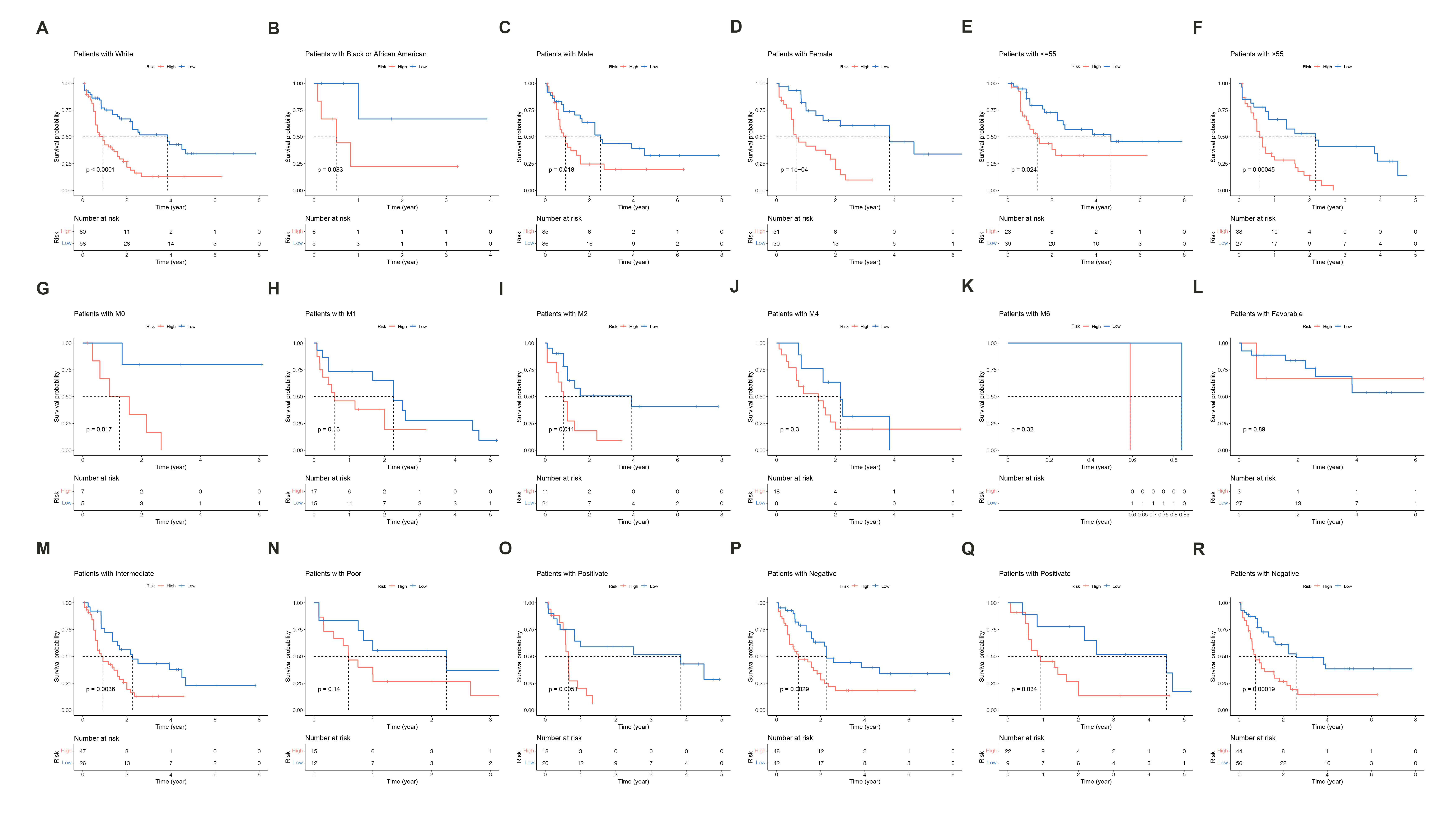

Supplement: Supplementary file 1 [file DataSheet1.ZIP › Supplementary Figure S5 .tif]

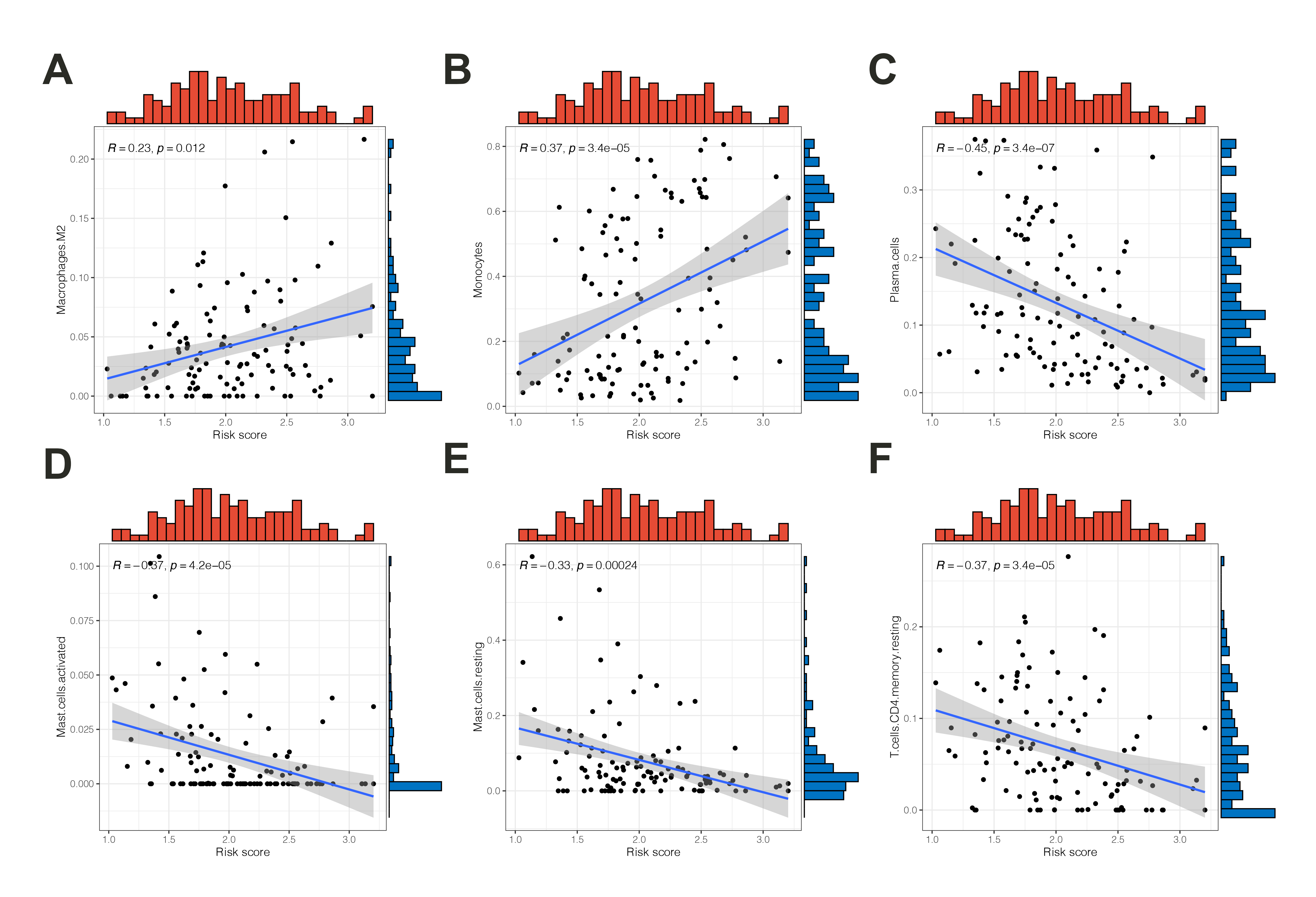

Supplement: Supplementary file 1 [file DataSheet1.ZIP › Supplementary Figure S6 .tif]

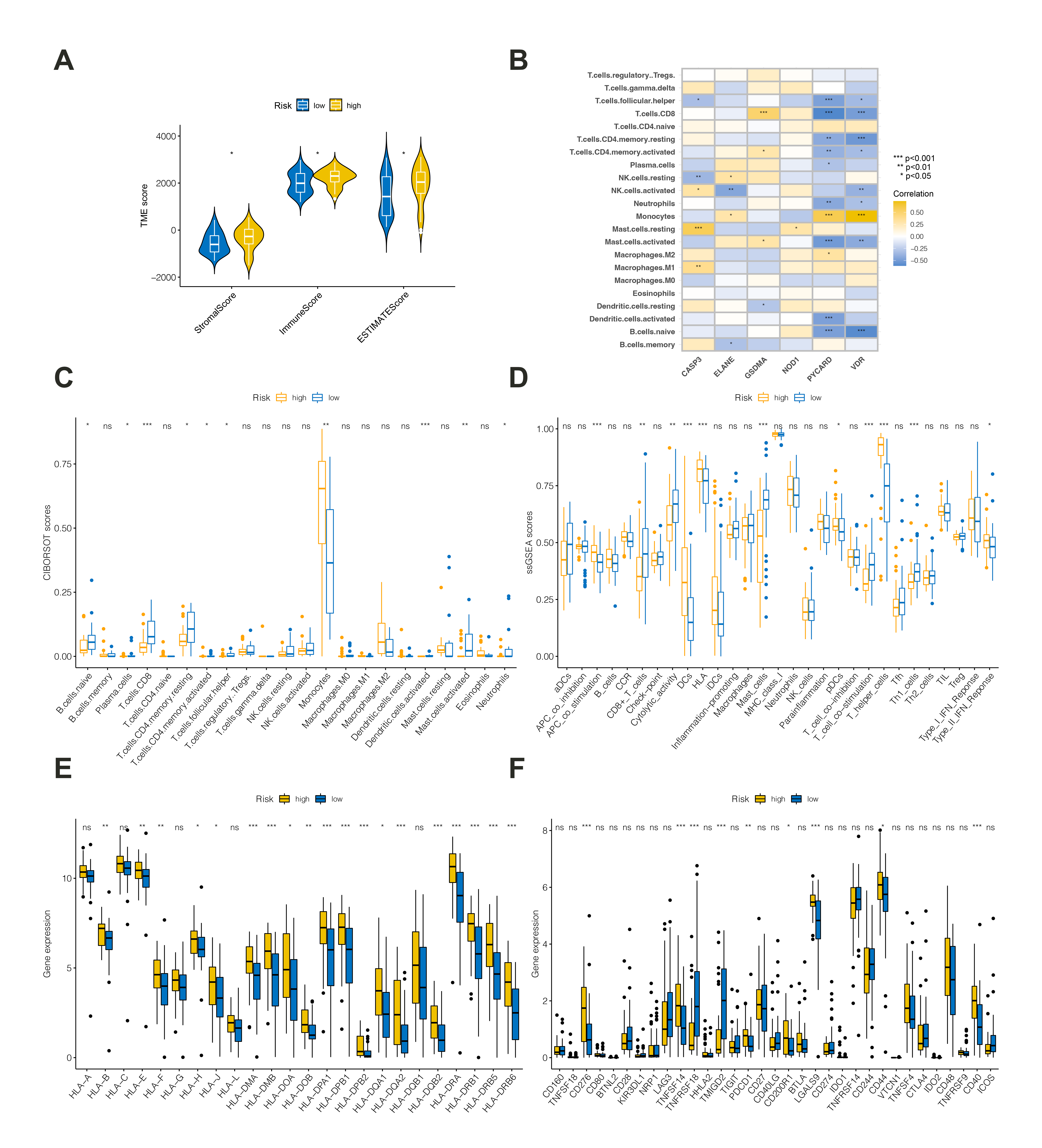

Supplement: Supplementary file 1 [file DataSheet1.ZIP › Supplementary Figure S7 .tif]

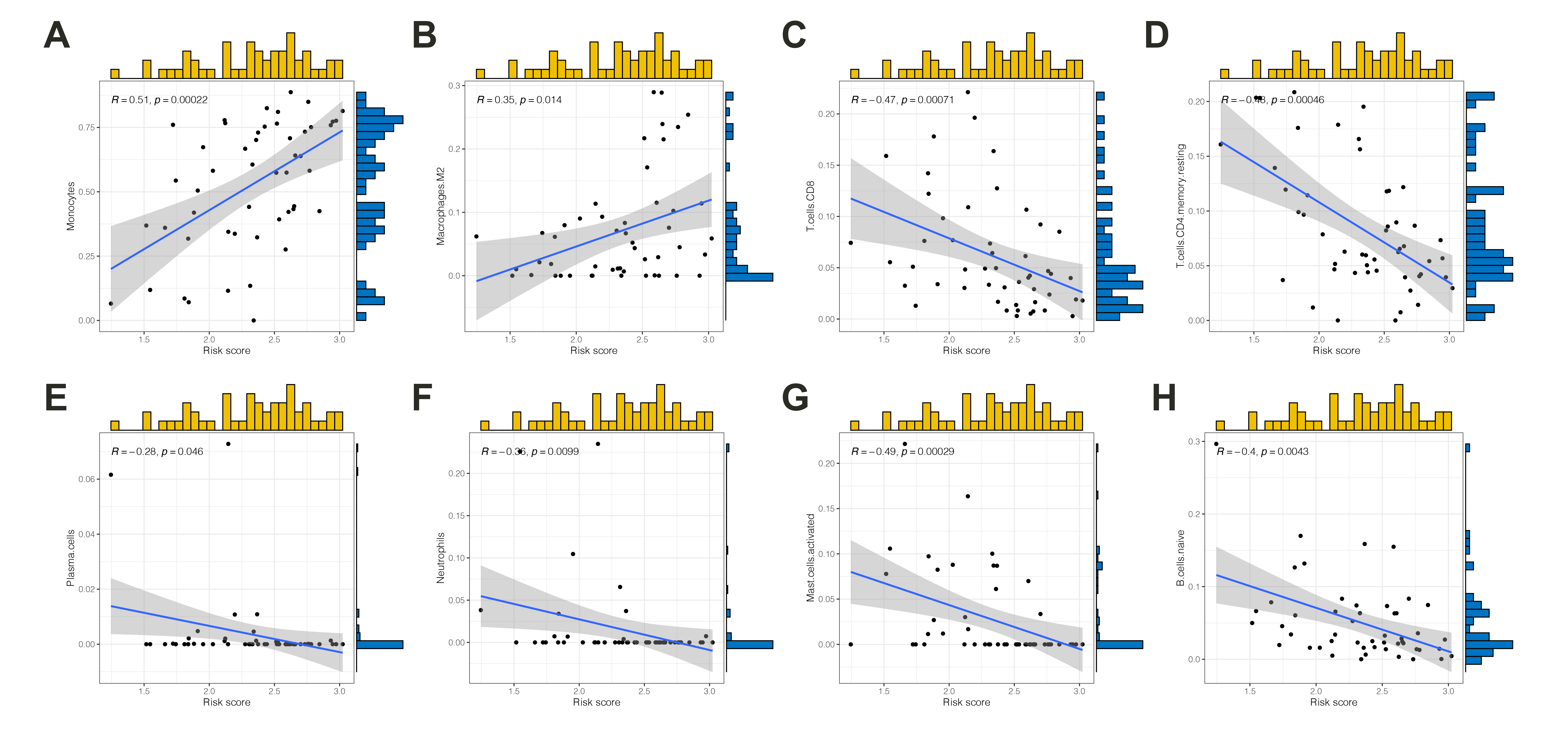

Supplement: Supplementary file 1 [file DataSheet1.ZIP › Supplementary Figure S8 .tif]
