# Supplementary material for: The pyroptosis-related gene signature predicts prognosis and reveals characterization of the tumor immune microenvironment in acute myeloid leukemia
Source: Front Pharmacol. 2022 Aug 10;13:951480. doi: 10.3389/fphar.2022.951480 (PMC9399441; doi:10.3389/fphar.2022.951480)
Supplement: Supplementary file 1 [file DataSheet1.ZIP › Supplementary Figure Legends.doc]

**Supplementary Figure Legends**

**Supplementary Figure 1.** Data sources and analysis flow chart.

**Supplementary Figure 2.** Unsupervised clustering of pyroptosis-related genes and Consensus matrix heatmaps for k = 2 (A), 4-9 (B-H). Cumulative distribution function curves (J) and Delta area (K) for k = 2-9 in the consistent clustering model.

**Supplementary Figure 3.** Prognostic study of the BeatAML cohort via the PRG signature model. (A) Risk scores and (B) survival status of AML patients. (C) PCA and (D) t-SNE analysis showing the different gene expression of samples. (E) Kaplan-Meier OS curves for high-risk and low-risk groups. (F) The 1-, 3-, and 5-year ROC curve to predict the survival status.

**Supplementary Figure 4.** Clinical evaluation of the PRG signature. The scatter diagram indicating that (A) NPM1 mutation, (B) cytogenetic risk, (C) subtypes, and (D) FAB classification were significantly associated with the risk score.

**Supplementary Figure 5.** Stratification analysis of the PRG score in AML. (A-B) Race (White and Black and African American). (C-D) Gender (male and female). (E-F) Age (<=55 years and >55 years). (G-K) FAB classification (M0, M1, M2, M4, and M6). (L-N) Cytogenetic risk (Favorable, Intermediate, and Poor). (O-P) NPM1 mutation (positive and negative). (Q-R) FLT3-ITD mutation (positive and negative).

**Supplementary Figure 6.** Spearman correlations between risk score and immune cell types in the TCGA cohort.

**Supplementary Figure 7.** Immune characteristics analysis in the BeatAML cohort. (A) Associations between risk score and both immune and stromal scores. (B) Correlations between the abundance of immune cells and 6 genes in the proposed signature. Comparison of the enrichment scores of (C) 22 kinds of immune cells and (D) 29 types of immune signatures between high- and low-risk groups. (E) Expression of HLA in the high and low-risk groups. (F) Expression of immune checkpoints in the high and low-risk groups. P values were showed as: ns not significant. *P = < 0.05; **P = < 0.01; ***P = < 0.001.

**Supplementary Figure 8.** Spearman correlations between risk score and immune cell types in the BeatAML cohort.
